# Agonist-antagonist muscular co-contraction improves rapid corrective responses

**DOI:** 10.1016/j.isci.2025.113122

**Published:** 2025-07-16

**Authors:** Daniel P. Armstrong, Kevin J. Deluzio, Stephen H. Scott

**Affiliations:** 1 Centre for Neuroscience Studies, Queen’s University, Kingston, ON, Canada; 2 Department of Mechanical and Materials Engineering, Queen’s University, Kingston, ON, Canada; 3 Department of Biomedical and Molecular Sciences, Queen’s University, Kingston, ON, Canada; 4 Department of Medicine, Queen’s University, Kingston, ON, Canada

**Keywords:** Biological sciences, Neuroscience, Biomechanics, Kinesiology

## Abstract

Muscular co-contraction (simultaneous activation of both agonist and antagonist muscle groups) has been observed to emerge across a range of challenging tasks. But is it beneficial? In this study we address this untested assumption by quantifying the performance benefits of muscular co-contraction when making rapid corrective responses to mechanical perturbations. Even small levels of co-contraction resulted in significant performance improvements compared to the relaxed condition in a target recapture task following a mechanical perturbation. Performance was also better when co-contracting compared to when a single muscle group was active prior to the perturbation. The performance benefits of co-contracting seem partially attributable to neural mechanisms where activation of both agonist and antagonist muscles allowed both groups to contribute to limb control via reflex responses. These findings highlight how co-contraction can greatly impact motor performance. Further, the benefit of co-contraction extends beyond just increased muscle activity providing instantaneous resistance to limb motion.

## Highlights

• Voluntary co-contraction improved performance when making rapid motor corrections
• Co-contraction had better performance than when a single muscle was pre-loaded
• Performance benefits are attributable to dual-control of agonist-antagonist muscles

## Graphical abstract

**Figure:**
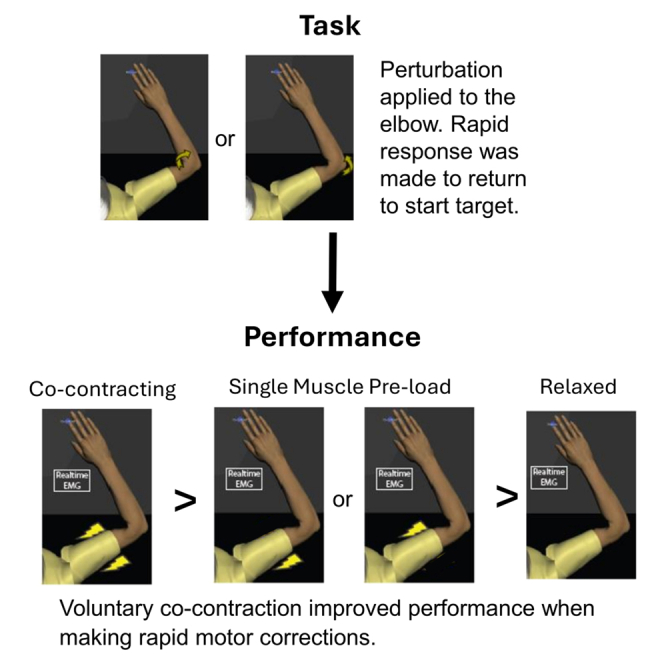

## Introduction

In our daily lives, we often experience unexpected disturbances in our environment, whether standing on a moving bus or carrying a pitcher of water. Muscular co-contraction (simultaneous activation of agonist and antagonist muscle groups) is commonly observed when performing difficult tasks. For example, co-contraction has been observed to improve joint control,^1^^,^^2^^,^^3^^,^^4^^,^^5^ regulate movement variability,^6^^,^^7^ and help learn unstable dynamics.^8^^,^^9^^,^^10^ While co-contraction commonly emerges across a range of challenging contexts, its benefits on improving rapid corrective responses have not been quantified beyond our preliminary observations.^11^

Co-contraction has been hypothesized to increase joint impedance (stiffness and damping) to instantaneously resist motion^12^ or oppose mechanical instability.^13^ An increase in muscle stiffness is partially a result of increased short-range stiffness when initially stretching an active muscle.^14^ This instantaneous contribution would circumvent the challenge of delays in sensory feedback to improve corrective responses to unexpected disturbances. The hypothesized impedance-based mechanism of co-contraction improving control is partially attributable to original experiments estimating muscle impedance >200 ms after a mechanical disturbance.^15^^,^^16^ This inadvertently attributes neural feedback responses to increases in impedance, as recognized in the original studies. While these initial observations were made ∼40 years ago, recent work continues to attribute co-contraction benefits on limb control to increases in impedance (i.e.,^17^^,^^18^). Contrary to these findings our recent work has found minimal differences in joint kinematics until >50 ms following a mechanical perturbation when contracting.^11^^,^^19^ This argues against the purely impedance-based mechanism of control previously proposed. The limited influence of co-contraction immediately following a disturbance reflects that limb inertia rather than stiffness and damping has the greatest influence on initial limb motion.^19^

We hypothesize that co-contraction may impact neural feedback mechanisms to improve responses to unexpected disturbances.^20^ This hypothesis is different from the concept of impedance-based neural control as the latter emphasizes the importance of setting muscle activation levels to alter muscle stiffness and damping to aid control. Co-contraction could increase the spinal stretch reflex response (i.e.,^20^^,^^21^^,^^22^^,^^23^^,^^24^^,^^25^^,^^26^^,^^27^) due to greater pre-perturbation muscle activity. Further, transcortical pathways may be modulated by co-contraction, as previously seen in a rodent model.^28^ This may be accomplished in practice by increasing the nervous system’s range of feedback responses.^29^ Finally, the simultaneous activation of agonist and antagonist muscle groups will reduce reciprocal inhibition,^30^^,^^31^ potentially facilitating a coordinated dual-control muscle response to unexpected disturbances with contributions from both opposing muscle groups.

**Figure 1:**
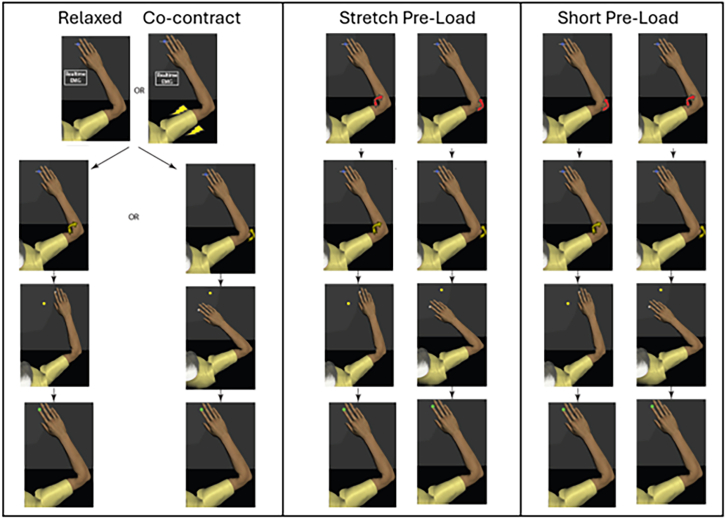
Overview of mechanical perturbation trials Different experimental conditions are pictured in different columns including: (left relaxed and co-contracting, middle) (stretched muscle pre-loaded, right) shortened muscle pre-loaded. A description of each component of a trial is described in each row: (top row) participants moved their fingertip to a spatial target when either relaxed (left), co-contracting to generate a level of muscle activity specified with real-time biofeedback (centre-left) or, resisting an applied background load (red arrow) that either extends (centre-right) or flexes (right) the arm. (Second row) A 5 Nm transient load (10 ms ramp, 50 ms duration) was applied to either flex or extend the elbow (yellow arrows). (Third row) participants made a corrective response to return to the target and hold their position as fast as possible. (Final row) the trial was successful if participants reacquired and stayed at the target within 500 ms. Successful trials were noted with a green target color, whereas unsuccessful trials displayed red.

The objective of this study was to quantify the performance benefits of muscular co-contraction on rapid corrective responses to mechanical perturbations. Secondly, we aimed to investigate changes in muscular activity patterns to infer neural contributions to co-contraction improving limb control. To investigate these objectives three experiments were completed where mechanical perturbations were applied to displace the elbow joint and participants were asked to reacquire the start target as accurately as possible (Figure 1). Pre-perturbation activity was systematically varied across experimental conditions to be either relaxed, co-contracting, resisting background load, and/or a combination of these factors. We found that muscular co-contraction improved corrective responses, with the simultaneous activation of both muscle groups providing greater performance than selectively activating a single muscle group.

## Results

### Experiment 1

**Figure 2:**
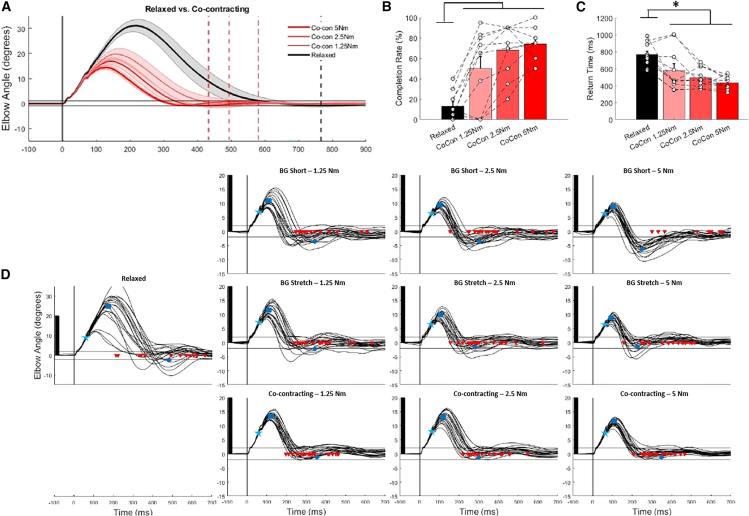
Visualization of time-series kinematics and performance metrics for co-contracting conditions, as well as sample participant data across all conditions (A) Time-series elbow displacement post-perturbation for relaxed and co-contracting conditions. Perturbation onset is shown with the vertical gray line, and mean return times are shown for each condition with dashed vertical lines. Solid black horizontal lines visualize the range for successful target recapture across conditions. Discrete comparisons of trial success rate and return times are shown for relaxed vs. co-contracting conditions. (B and C) All data are reported as mean +/− standard error. (D) Sample kinematics following perturbation for an exemplar subject across relaxed, co-contracting, and resisting background (BG) load conditions. Perturbation onset is denoted with the vertical gray line, and the successful target recapture range is visualized with horizontal black lines. Return times are shown with red arrows for each trial. Mean displacement 50 ms post-perturbation (cyan star), maximum displacement (blue square) and maximum overshoot (blue diamond) at their respective times are shown with red symbols. Note: The *y* axis scales differ between the Relaxed and remaining conditions. The black rectangles on each subplot are consistent in size to visualize differences in scale. The peaks of some curves are cut off to maintain figure resolution.

Across all experimental conditions mechanical perturbations applied during postural task resulted in a bell-shaped kinematic profile generating a rapid corrective response to return the joint to its initial position and reacquire the start target (Figures 2D and S1). The within-participant mean curves were retained as dependent measures, as well as return time and completion rates.

We first compared differences in corrective responses between relaxed and co-contracting conditions. In general, co-contracting trials had reduced peak angular displacement and resulted in a faster return time to the spatial target (Figure 2A). Comparisons of discrete performance variables confirmed that successful trial completion rates were greater (*p* < 0.001, ꞃ^2^ = 0.752, F(3,27) = 27.3) and return times were reduced (*p* < 0.001, ꞃ^2^ = 0.632, F(3,27) = 15.4) when co-contracting compared to the relaxed condition (Figures 2B and 2C). Notably, return times significantly decreased from the relaxed condition even for the lowest level of co-contraction in 7/10 participants (Table S1). While there was a large step in performance between the relaxed condition and the lowest level of co-contraction, there were further corresponding improvements in performance at increasing co-contraction levels with mean return times further decreasing as the co-contraction levels increased. However, while a visual trend of increasing performance was observed as a function of increasing co-contraction levels no post-hoc differences supporting this observation were found.

With the performance benefits of co-contraction relative to relaxed conditions shown, we then aimed to compare corrective responses between co-contracting and when a single muscle is pre-loaded. Having both stretched and shortened muscle group pre-load conditions was important as both can mechanically contribute to corrective responses due to the force-velocity relationship of muscle.^32^ To test these findings’ generalizability, these comparisons were made with a total of 5 and 2.5 Nm of muscle activity dispersed across the muscle groups.

**Figure 3:**
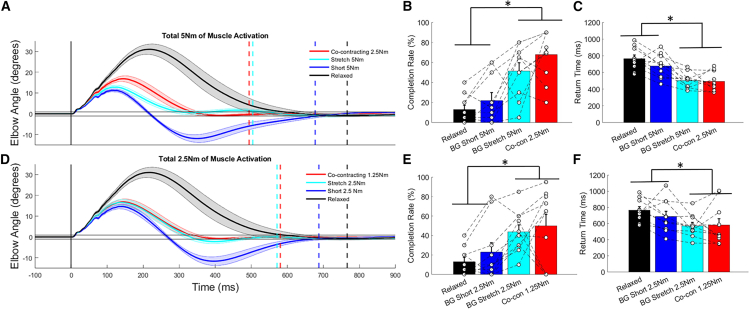
Time-series elbow displacement following perturbation and discrete performance measures when comparing either a total of 5 Nm or 2.5 Nm of muscle activity prior to perturbation conditions Time-series elbow displacement following perturbation when comparing (A) a total of 5 Nm and (D) 2.5 Nm of muscle activity prior to perturbation. Relaxed conditions are included on each plot for reference. Perturbation onset is shown with the vertical gray line, and mean return times are shown for each condition with dashed vertical lines. Solid black horizontal lines visualize the range for successful target recapture across conditions. Discrete comparisons of trial success rate and return times are shown for the total of 5 Nm (B and C), and 2.5 Nm (E and F) of muscle activation conditions. Mean data are shown with solid lines and standard error is shown by the shaded regions and significant post-hoc differences between groups are noted with “∗” for discrete measures. In the discrete data plots, all data are reported as mean +/− standard error.

Differences in time-series kinematics were observed across relaxed, stretched muscle pre-loaded, shortened muscle pre-load, and co-contracting conditions (Figures 3A and 3D). In particular, overshoot-related detriments of pre-loading the shortened muscle are observed, influencing performance. At both the total of 5 and 2.5 Nm of activation conditions there is larger overshoot when the shortened muscle is pre-loaded compared to all other experimental conditions (Table 1). Even though maximum displacement was reduced in the shortened muscle pre-load condition compared to co-contracting and relaxed conditions, this did not lead to improved performance (Table 1). As a supplementary comparison, maximum displacement did not differ between when the stretched muscle pre-load and co-contracting conditions for the same level of stretched muscle activity (1.25 Nm (*p* = 0.540, d = 0.201, t(9) = 2.2), 2.5 Nm (*p* = 0.732, d = 0.114, t(9) = 1.5), and 5 Nm (*p* = 0.427, d = 0.273, t(9) = 3.3)).

**Table 1:** Group means and standard deviations of discrete kinematic measures for the experimental conditions investigated in experiment 1

	Relaxed	Co-contracting	Stretched pre-load	Shortened pre-load	Statistical testing outcomes	Post-hoc differences
**Total 5 Nm activation**						

Displacement at 50 ms (deg.)	9.57 (1.23)	8.49 (1.09)	7.60 (1.12)	7.03 (0.85)	*p* < 0.001, ꞃ^2^ = 0.461, F(3,27) = 7.7	Rel > Co > St > Sh
Displacement at 100 ms (deg.)	17.14 (3.12)	14.07 (2.39)	12.12 (2.33)	10.78 (1.85)	*p* < 0.001, ꞃ^2^ = 0.516, F(3,27) = 9.6	Rel > Co > Sh > St
Max Disp. (deg.)	33.01 (7.87)	17.84 (4.77)	14.02 (3.49)	14.52 (4.62)	*p* < 0.001, ꞃ^2^ = 0.693, F(3,27) = 20.3	Rel > Co > St, Sh
Max OS (deg.)	5.64 (2.17)	2.82 (1.64)	3.08 (1.61)	12.88 (5.66)	*p* < 0.001, ꞃ^2^ = 0.645, F(3,27) = 16.4	Sh > Rel > Co, St

**Total 2.5 Nm Activation**						

Displacement at 50 ms(deg.)	9.57 (1.23)	8.87 (1.23)	8.51 (1.40)	7.68 (0.96)	*p* < 0.001, ꞃ^2^ = 0.262, F(3,27) = 3.2	Rel > Co, St > Sh
Displacement at 100 ms (deg.)	17.14 (3.12)	15.12 (3.06)	14.25 (3.03)	12.44 (2.34)	*p* < 0.001, ꞃ^2^ = 0.272, F(3,27) = 3.4	Rel > Co, Sh > St
Max Disp. (deg.)	33.01 (7.87)	21.54 (7.48)	17.31 (4.23)	16.94 (4.81)	*p* < 0.001, ꞃ^2^ = 0.544, F(3,27) = 10.7	Rel > Co, St, Sh
Max OS (deg.)	5.64 (2.17)	3.64 (2.95)	4.95 (2.05)	13.01 (6.14)	*p* < 0.00, ꞃ^2^ = 0.525, F(3,27) = 9.9	Sh > Rel, Co, St

Statistical test outcomes from one-way repeated measures ANOVAs and corresponding post-hoc differences are reported.

Note: Rel, relaxed; co, co-contracting; St, stretched pre-load; Sh, shortened pre-load.

When the stretched muscle was pre-loaded there was comparable performance to when co-contracting. The greater activation of the stretched muscle limited the maximum displacement compared to when co-contracting (Table 1). Collectively, this resulted in the greatest completion rates in the co-contracting and stretched muscle pre-load conditions at both a total of 5 Nm (*p* < 0.001, ꞃ^2^ = 0.517, F(3,27) = 9.6; Figure 3B) and 2.5 Nm (*p* = 0.002, ꞃ^2^ = 0.256, F(3,27) = 3.1; Figure 3E) of activation. Corresponding differences were seen in return times at 5 Nm activation (*p* < 0.001, ꞃ^2^ = 0.490, F(3,27) = 8.6; Figure 2C) and 2.5 Nm of activation (*p* = 0.008, ꞃ^2^ = 0.172, F(3,27) = 1.9; Figure 2F). However, no post-hoc differences in return times were seen at the 2.5 Nm activation level.

### Experiment 2

The benefits of co-contraction relative to when only a stretched muscle was pre-loaded was shown in experiment 1, but this comparison did not differentiate between pre-loading stretched and shortened muscle groups. To investigate this, perturbations were applied following pre-loading a single muscle group. If the perturbation direction was the same as the direction of applied background loads it is referred to as the “stretched muscle pre-load” condition, while opposite directions are the “shortened muscle pre-load” condition.

**Figure 4:**
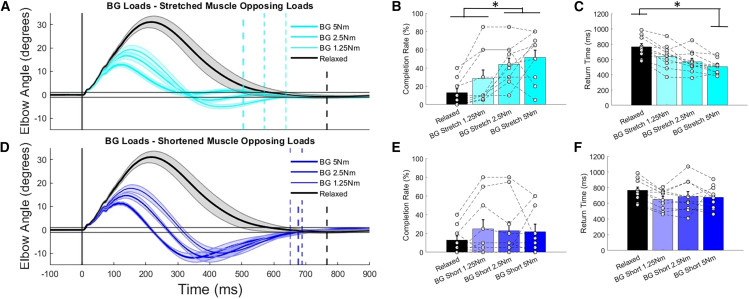
Time-series elbow displacement following perturbation and discrete performance measures when comparing either stretched or shortened muscles were pre-loaded prior to perturbation Time-series elbow displacement following perturbation when comparing across relaxed and (A) when stretched muscles are opposing loads, and (D) when shortened muscles are opposing loads. Perturbation onset is shown with the vertical gray line, and mean return times are shown for each condition with dashed vertical lines. Solid black horizontal lines visualize the range for successful target recapture across conditions. Discrete comparisons of trial success rate and return times are shown for relaxed vs. stretched muscle resisting background loads (B and C) and shortened muscle resisting background loads (E and F) conditions. Mean data are shown with solid lines and standard error is shown by the shaded regions and significant post-hoc differences between groups are noted with “∗” for discrete measures. In the discrete data plots, all data are reported as mean +/− standard error.

When the stretched muscle was pre-loaded prior to perturbation, there were differences in time-series kinematics from the relaxed condition with maximum angular displacement again reduced (Figure 4A). Again, the largest difference in time-series kinematics was from the relaxed to pre-loaded conditions (similar to results across the co-contracting conditions). Unlike when co-contracting, there was overshoot when reacquiring the target observed when the stretched muscle was pre-loaded, with the lowest activation level having the greatest overshoot (Table 2). Pre-loading the stretched muscle had observed performance benefits as trial completion rates were significantly greater at the 2.5 and 5 Nm pre-loading levels compared to the 1.25 Nm pre-load and relaxed conditions (*p* < 0.001, ꞃ^2^ = 0.580, F(3,27) = 12.4; Figure 4B). Finally, the lowest return times across this condition were observed when pre-loading the stretched muscle to 5 Nm as they were significantly lower than return times in the relaxed condition (*p* < 0.001, ꞃ^2^ = 0.741, F(3,27) = 25.7; Figure 4C).

**Table 2:** Group means and standard deviations of discrete kinematic measures for the experimental conditions investigated in experiment 2

Stretched muscle pre-load	Relaxed	Stretch 1.25 Nm	Stretch 2.5 Nm	Stretch 5 Nm	Statistical testing outcomes	Post-hoc differences
Displacement at 50 ms (deg.)	9.57 (1.23)	8.80 (1.49)	8.51 (1.40)	7.60 (1.12)	*p* < 0.001, ꞃ^2^ = 0.242, F(3,27) = 2.9	Rel > St1.25 > St2.5 > St5
Displacement at 100 ms (deg.)	17.14 (3.12)	15.26 (3.32)	14.25 (3.03)	12.12 (2.33)	*p* < 0.001, ꞃ^2^ = 0.298, F(3,27) = 3.8	Rel > St1.25 > St2.5 > St5
Max Disp. (deg.)	33.01 (7.87)	20.14 (5.37)	17.31 (4.23)	14.02 (3.49)	*p* < 0.001, ꞃ^2^ = 0.660, F(3,27) = 17.4	Rel > St1.25 > St2.5 > St5
Max OS (deg.)	5.64 (2.17)	7.90 (2.56)	4.95 (2.05)	3.08 (1.61)	*p* < 0.001, ꞃ^2^ = 0.424, F(3,27) = 6.6	St1.25 > Rel, St2.5 > St5

Shortened muscle pre-load	Relaxed	Short 1.25 Nm	Short 2.5 Nm	Short 5 Nm	Statistical testing outcomes	Post-hoc differences
Displacement at 50 ms (deg.)	9.57 (1.23)	8.11 (1.11)	7.68 (0.96)	7.03 (0.85)	*p* < 0.001, ꞃ^2^ = 0.477, F(3,27) = 8.2	Rel > Sh1.25 > Sh2.5 > Sh5
Displacement at 100 ms (deg.)	17.14 (3.12)	13.76 (2.67)	12.44 (2.34)	10.78 (1.85)	*p* < 0.001, ꞃ^2^ = 0.485, F(3,27) = 8.5	Rel > Sh1.25 > Sh2.5 > Sh5
Max Disp. (deg.)	33.01 (7.87)	18.93 (4.61)	16.94 (4.81)	14.52 (6.62)	*p* < 0.001, ꞃ^2^ = 0.672, F(3,27) = 18.4	Rel > Sh1.25 > Sh2.5 > Sh5
Max OS (deg.)	5.64 (2.17)	11.93 (5.31)	13.01 (6.14)	12.88 (5.66)	*p* < 0.001, ꞃ^2^ = 0.293, F(3,27) = 3.7	Rel > Sh1.25, Sh2.5, Sh5

Statistical test outcomes from one-way repeated measures ANOVAs and corresponding post-hoc differences are reported.

Note: Rel, relaxed; Co, co-contracting; St, stretched pre-load; Sh, shortened pre-load. The numbers 5, 2.5, and 1.25 refer to the magnitude of muscle group activation in Nm.

When the shortened muscle was pre-loaded there was again a clear difference between the relaxed and pre-loaded conditions in time-series kinematics (Figure 4D). However, the time-series kinematics when the shortened muscle was pre-loaded notably differed from the co-contracting and stretched muscle pre-load condition as there was consistent and greater target overshoot, which in turn, reduced return times to the start target (Table 2). This observed target overshoot negated the benefits of the pre-loaded shortened muscle reducing maximum angular displacement as no significant differences in either completion rate (*p* = 0.230, ꞃ^2^ = 0.145, F(3,27) = 1.5; Figure 4E) or return time (*p* = 0.115, ꞃ^2^ = 0.194, F(3,27) = 2.2; Figure 4F) were observed.

**Figure 5:**
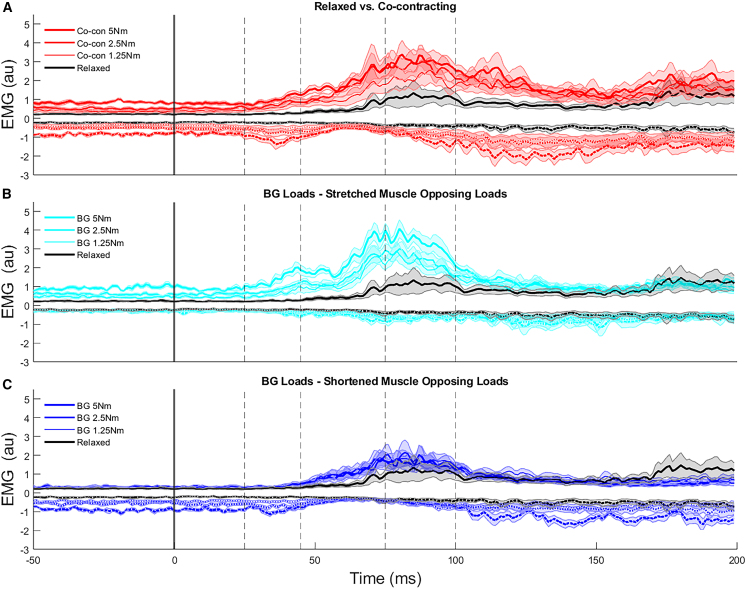
Time-series EMG of the stretched (solid lines, positive values) and shortened (dashed lines, negative values) muscles following perturbation across relaxed, co-contracting, and single muscle pre-load conditions Time-series EMG of the stretched (solid lines, positive values) and shortened (dashed lines, negative values) muscles following perturbation across relaxed and (A) co-contracting conditions, (B) when stretched muscles are opposing loads, and (C) when shortened muscles are opposing loads. Perturbation onset is shown with the vertical gray lines. Mean data are shown with solid lines and standard error is shown by the shaded regions.

The observed performance and time-series kinematic differences across conditions are likely attributed to corresponding differences in EMG. In the stretched muscle pre-load condition greater activation was observed in the stretched muscle in both short- and long-latency epochs, with no apparent time-series differences in the shortened muscle (Figure 5B). Epochs were defined based on time following perturbation (R1 = 20–45 ms, R2 = 45–75 ms and R3 = 75–105 ms; epoch definitions from^33^) for both lengthened (R#L) and shortened (R#S) muscle groups. Statistical testing confirmed significantly greater stretched muscle activity in the R2L *(p* < 0.001, ꞃ^2^ = 0.771, F(3,27) = 30.3) and R3L epochs (*p* < 0.001, ꞃ^2^ = 0.547, F(3,27) = 10.8) with no corresponding differences in the R1L *(p* = 0.003, ꞃ^2^ = 0.402, F(3,27) = 6.1), R1S *(p* = 0.129, ꞃ^2^ = 0.186, F(3,27) = 2.0), R2S *(p* = 0.010, ꞃ^2^ = 0.340, F(3,27) = 4.6) and R3S (*p* = 0.010, ꞃ^2^ = 0.336, F(3,27) = 4.5) time epochs (Figure S2).

Opposite trends were observed in the shortened muscle pre-load condition. There was a depression in shortened muscle EMG through the long-latency time epoch (∼45–105 ms; Figure 5C). When expressing these EMG levels relative to pre-perturbation activity there was a significant difference in the R1S, R2S, and R3S epochs (*p* < 0.002, ꞃ^2^ > 0.427, F(3,27) > 6.7; Figure S2), supporting the observed time-series differences. In the shortened pre-loaded condition, significant differences in stretched muscle activity were also observed compared to the relaxed condition for the R2L (*p* < 0.001, ꞃ^2^ = 0.474, F(3,27) = 8.1), but not the R1L (*p* = 0.025, ꞃ^2^ = 0.289, F(3,27) = 3.6) and R3L *(p* = 0.133, ꞃ^2^ = 0.184, F(3,27) = 2.0) epochs (Figure S2).

When co-contracting, the changes in stretched and shortened muscle activity observed in their respective pre-loading conditions were seen. These changes included both greater short- and long-latency stretched muscle activity, and a concurrent depression in shortened long-latency muscle activity when co-contracting (Figure 5A). Statistical testing confirmed these observed effects with significant increases in muscle activity in R2L (*p* < 0.001, ꞃ^2^ = 0.598, F(3,27) = 13.4) and R3L (*p* < 0.001, ꞃ^2^ = 0.558, F(3,27) = 11.3), but not the R1L (*p* = 0.010, ꞃ^2^ = 0.338, F(3,27) = 4.6) epoch. However, in the shortened muscle, no significant differences were observed in any of the R1S (*p* = 0.025, ꞃ^2^ = 0.289, F(3,27) = 3.6), R2S (*p* = 0.211, ꞃ^2^ = 0.151, F(3,27) = 1.6), or R3S (*p* = 0.285, ꞃ^2^ = 0.129, F(3,27) = 1.3) time epochs (Figure S2).

### Experiment 3

In the final experiment we investigated the relative neural contributions of co-contraction on rapid corrective responses when the level of muscle activity was not consistent between the stretched and shortened muscle groups. In this experiment pre-perturbation activity was systematically modified to be a 2:1 ratio between elbow flexor and extensor muscle groups. This was done at two magnitudes, either having 5 and 2.5 Nm or 2.5 and 1.25 Nm of activation in the opposing muscle groups. In addition to making these comparisons to a relaxed condition, a reference background load condition was also included when a single muscle was pre-loaded to the magnitude of difference between the stretched and shortened muscle groups at the higher activity level.

**Figure 6:**
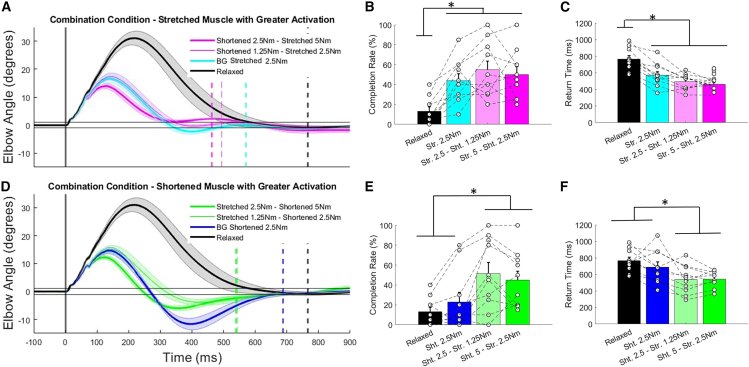
Time-series elbow displacement following perturbation and discrete performance measures when comparing either combined muscle activation conditions where either the stretched or shortened muscles were pre-loaded to a higher magnitude prior to perturbation Time-series elbow displacement following perturbation in combination conditions when (A) stretched muscle activity is greater than shortened muscle activity and (B) shortened muscle activity is greater than stretched muscle activity. Relaxed and an exemplar background load condition data are included on each plot for reference. Perturbation onset is shown with the vertical gray line, and mean return times are shown for each condition with dashed vertical lines. Solid black horizontal lines visualize the range for successful target recapture across conditions. Discrete comparisons of trial success rate and return times are shown for the stretched muscle activity is greater than shortened muscle activity (B and C), and shortened muscle activity is greater than stretched muscle activity (E and F) conditions. Mean data are shown with solid lines and standard error is shown by the shaded regions and significant post-hoc differences between groups are noted with “∗” for discrete measures. In the discrete data plots, all data are reported as mean +/− standard error.

Consistent with previous comparisons there was again a large discrepancy between time-series kinematics when relaxed compared to any condition with pre-activation of muscle. When the muscle activity was greater in the shortened muscle there was again overshoot when returning to the target. However, with some activation of the stretched muscle pre-perturbation this overshoot was reduced compared to the shortened muscle pre-load condition (Figure 6A; Table 3). With some activation of the stretched muscle groups limiting overshoot there was significantly improved success rates (*p* < 0.001, ꞃ^2^ = 0.605, F(3,27) = 13.8; Figure 6B) and reduced return times (*p* < 0.001, ꞃ^2^ = 0.585, F(3,27) = 12.7; Figure 6C) with greater activation in the shortened muscle group compared to relaxed and shortened muscle pre-load conditions.

**Table 3:** Group means and standard deviations of discrete kinematic measures for the experimental conditions investigated in experiment 3

Shortened greater activation	Relaxed	Short 5Nm, Stretch 2.5	Short 2.5Nm, Stretch 1.25	Shortened pre-load 2.5 Nm	Statistical testing outcomes	Post-hoc differences
Displacement at 50 ms (deg.)	9.57 (1.23)	7.58 (0.93)	7.89 (1.08)	7.68 (0.96)	*p* < 0.001, ꞃ^2^ = 0.391, F(3,27) = 5.8	Rel > Sh5St2.5, Sh2.5St1.25, Sh
Displacement at 100 ms (deg.)	17.14 (3.12)	12.88 (2.32)	14.53 (2.62)	12.44 (2.34)	*p* < 0.001, ꞃ^2^ = 0.412, F(3,27) = 6.3	Rel > Sh2.5St1.25, Sh > Sh5St2.5
Max Disp. (deg.)	33.01 (7.87)	12.90 (2.43)	16.22 (4.50)	16.94 (4.81)	*p* < 0.001, ꞃ^2^ = 0.713, F(3,27) = 22.2	Rel > Sh2.5St1.25, Sh > Sh5St2.5
Max OS (deg.)	5.64 (2.17)	6.92 (2.63)	6.24 (3.60)	13.01 (6.14)	*p* < 0.001, ꞃ^2^ = 0.385, F(3,27) = 5.6	Sh > Rel, Sh5St2.5, Sh2.5St1.25

Stretched greater activation	Relaxed	Stretch 5Nm, Short 2.5	Stretch 2.5Nm, Short 1.25	Stretched Pre-load 2.5 Nm	Statistical testing outcomes	Post-hoc differences
Displacement at 50 ms (deg.)	9.57 (1.23)	8.18 (1.24)	8.75 (1.30)	8.51 (1.40)	*p* < 0.001, ꞃ^2^ = 0.153, F(3,27) = 1.6	Rel > St5Sh2.5, St2.5Sh1.25, St
Displacement at 100 ms (deg.)	17.14 (3.12)	11.51 (1.86)	12.82 (2.41)	14.25 (3.03)	*p* < 0.001, ꞃ^2^ = 0.356, F(3,27) = 4.9	Rel > St2.5Sh1.25 > St5Sh2.5, St
Max Disp. (deg.)	33.01 (7.87)	14.82 (3.53)	18.01 (4.11)	17.31 (4.23)	*p* < 0.001, ꞃ^2^ = 0.687, F(3,27) = 19.7	Rel > St2.5Sh1.25, St > St5Sh2.5,
Max OS (deg.)	5.64 (2.17)	3.57 (3.20)	2.77 (1.62)	4.95 (2.05)	*p* = 0.057, ꞃ^2^ = 0.212, F(3,27) = 2.4	N/A

Statistical test outcomes from one-way repeated measures ANOVAs and corresponding post-hoc differences are reported.

Note: Rel, relaxed; co, co-contracting; St, stretched pre-load; Sh, shortened pre-load. The numbers 5, 2.5, and 1.25 refer to the magnitude of muscle group activation in Nm.

With greater pre-perturbation activity in the stretched muscle there were similarities in the time-series kinematics compared to when just the stretched muscle was pre-loaded (Figure 6D). This is supported by no significant differences in displacement 100 ms post-perturbation when there was 2.5 Nm greater activation in the stretched muscle compared to the shortened muscle, with or without shortened muscle activity (Table 3). With some activity in the shortened muscle there was resultant lower mean return times compared to the stretched muscle pre-load condition due to improved accuracy when returning to the target. However, significant differences in target success rate (*p* < 0.001, ꞃ^2^ = 0.601, F(3,27) = 13.5; Figure 6E) and return time (*p* < 0.001, ꞃ^2^ = 0.772, F(3,27) = 30.5; Figure 6F) were only seen relative to the relaxed condition.

Collectively, the findings in this third experiment reiterate the benefit of having both agonist and antagonist muscle groups active prior to perturbation leads to performance benefits. By having both muscles active there is no reciprocal inhibition of an opposing muscle groups, allowing for the dual-control of the limb. Conversely, muscle impedance seems to primarily be related to limiting maximum displacement of the limb as seen with maximum displacement not significantly differing between conditions when just a single muscle group is pre-loaded or when there is also 1.25 Nm of activity in the opposing muscle group (Table 3). This suggests that while impedance does have an influence on the displacement of the limb, it is not the primary factor influencing success on this task.

**Figure 7:**
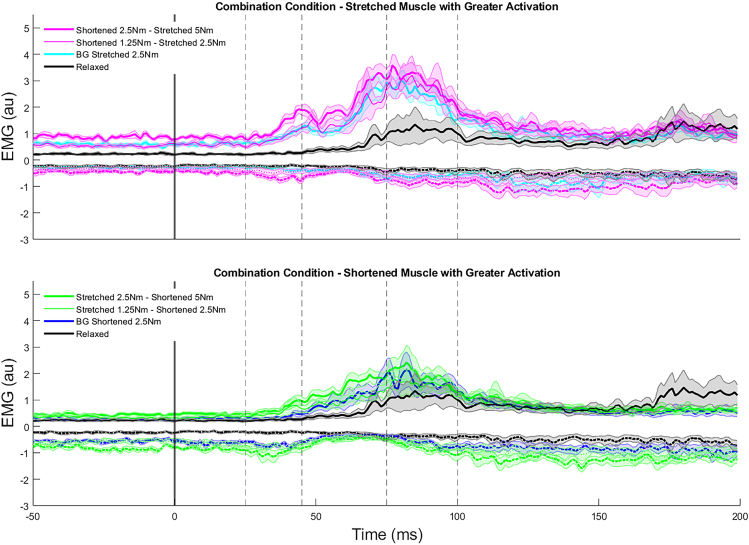
Time-series EMG of the stretched (solid lines, positive values) and shortened (dashed lines, negative values) following perturbation when stretched muscle activity is greater than shortened muscle activity and shortened muscle activity is greater than stretched muscle activity Time-series EMG of the stretched (solid lines, positive values) and shortened (dashed lines, negative values) following perturbation when (top) stretched muscle activity is greater than shortened muscle activity and (bottom) shortened muscle activity is greater than stretched muscle activity. Relaxed and an exemplar background load condition are included on each plot for reference. Perturbation onset is shown with the vertical gray lines. Mean data are shown with solid lines and standard error is shown by the shaded regions on time-series plots.

The improved kinematic performance when both muscles are asymmetrically active prior to perturbation are again likely attributable to EMG differences. When the shortened muscle had greater pre-perturbation activity the most pronounced EMG differences were in long-latency depression of shortened muscle activity (Figure 7A). These time-series differences were reflected in the reduced R2S activity when the shortened muscles had greater activation (*p* < 0.001, ꞃ^2^ = 0.719, F(3,27) = 23.0; Figure S3). When there was greater stretched muscle activity the most notable feature of the time-series EMG curves is the increases in short- and long-latency stretched muscle activity, with some depression of the shortened muscle group in the long-latency epochs (Figure 7B). This was observed in both the R1L (*p* < 0.001, ꞃ^2^ = 0.578, F(3,27) = 12.3), R2L (*p* < 0.001, ꞃ^2^ = 0.671, F(3,27) = 18.3), and R3L (*p* = 0.002, ꞃ^2^ = 0.421, F(3,27) = 6.5) time epochs (Figure S3).

Across all experiments EMG activity was interpreted to explain performance differences. With the goal of relating pre-perturbation activity to performance, a final comparison was made correlating pre-perturbation stretched and shortened muscle activity to return time across conditions. Unsurprisingly, stretched muscle activity was significantly negatively correlated to return times (r = 0.487). Significant negative associations were also seen from pre-perturbation shortened muscle activity to return time (r = 0.234), albeit with a smaller effect size.

### Relating short-latency muscle activity to kinematics

Increased short-latency stretched muscle activity in the co-contracting, stretched muscle pre-load and combination conditions likely reduced maximum displacement. This is supported by the strongest associations being observed in the co-contracting (r = 0.380 ± 0.203, β = −5.11 ± 6.77, significant for 6/10 participants), stretched muscle pre-load condition (r = 0.472 ± 0.229, β = −3.43 ± 3.57, significant for 8/10 participants) and in the greater stretched muscle activity combination condition (r = 0.397 ± 0.174, β = −2.45 ± 3.09, significant for 6/10 participants). Notably, the directionality of these associations highlight reductions in maximum displacement as a function of short-latency activity (Tables S2 and S3). Conversely, correlations were weaker in both the shortened pre-load condition (r = 0.243 ± 0.113, β = 0.51 ± 5.63, significant for 3/10 participants) and in the greater shortened muscle activity combination condition (r = 0.316 ± 0.227, β = −3.74 ± 5.54, significant for 2/10 participants).

Greater stretched muscle short-latency activity in the shortened muscle pre-load condition was associated with greater target overshoot (Figure S4A; Tables S4 and S5). When pre-loading the shortened muscle, significant associations were seen for 5/10 participants with a positive direction of association (r = 0.234 ± 0.133, β = 2.89 ± 8.91). Conversely, a negative association was seen when regressing the stretched muscle pre-load condition to overshoot (r = 0.304 ± 0.167, β = −1.80 ± 2.43, significant for 5/10 participants; Figure S4B). Meanwhile, fewer significant correlations were seen in the co-contracting (r = 0.190 ± 0.150, β = −1.37 ± 1.89, significant for 4/10 participants), greater stretched muscle activity (r = 0.173 ± 0.127, β = −0.98 ± 2.62, significant for 1/10 participants) and greater shortened muscle activity (r = 0.317 ± 0.204, β = −2.03 ± 4.06, significant for 2/10 participants) combination conditions. These findings highlight that when the shortened muscle was pre-loaded the subsequent inhibition resulted in the stretched muscle activity generating target overshoot.

## Discussion

An untested assumption has been that motor performance improves when co-contracting antagonist muscle groups during difficult or novel tasks. By explicitly controlling co-contraction, our results highlight that even low levels of co-contraction can greatly improve motor performance, generating faster and more accurate corrective responses to mechanical disturbances compared to baseline performance.^11^ Critically, we now show here that performance is not just a product of the level of muscle activity, as co-contracting still provides better performance than when there is even double the level of activity in a single muscle group. One benefit when co-contracting is allowing a dual-control strategy of agonist and antagonist muscle groups to improve task performance. This argues against increases in muscle impedance being the only benefit of co-contraction on motor performance. The present study also highlights that even a small amount of muscle co-contraction (amount of activity in elbow flexors less than required to hold a pound of butter in your hand with the forearm horizontal) results in an abrupt improvement in performance compared to the baseline condition.

The performance benefits of co-contraction are observed in the challenging behavioral paradigm used in this study. Previous studies have used stepwise mechanical perturbations to explore task-based performance such as when perturbing the upper limb in goal-directed reaching (i.e.,^34^^,^^35^^,^^36^^,^^37^^,^^38^^,^^39^^,^^40^). However, the high magnitude, short duration perturbation pulses used in the present study led to faster initial motion and likely posed a more difficult challenge to the motor system. This paradigm requires multiple responses as both the perturbation onset and offset will influence limb displacements and motor responses,^34^ requiring a complex response to reacquire the target within the 500 ms time limit. Even though this task was designed to test the limits of the motor system, co-contraction was an effective strategy to improve task performance.

Many studies identify that changes in mechanical properties of muscle when co-contracting are involved in improving our ability to counter mechanical disturbances.^8^^,^^9^^,^^41^^,^^42^ Increases in co-contraction influence the stiffness and damping of the muscle, resisting joint motion. In addition to the proportional increase in muscle stiffness and damping with contraction, greater short-range muscle stiffness is observed following contraction,^14^ which has been argued to provide added benefit to counter mechanical disturbances.^43^ Collectively, these mechanical features are thought to provide zero-delay responses to perturbations.^44^ However, the instantaneous impact of muscle impedance is relatively modest prior to neural responses (i.e., prior to 50 ms) as this initial motion is dominated by the inertial properties of the limb.^19^ Thus, the benefit provided by muscle impedance largely occurs after 50 ms when neural responses also participate. Simulations suggest that the impedance properties of muscle can counter motor corrections but predict corrective responses that are slower than observed for human motor corrections.^11^ Combined, the previous findings as well as experimental evidence in the current study suggest that the benefits of co-contraction on performance extend beyond just muscle mechanics. If increased impedance was the biggest determinant of performance, then similarities in time-series kinematics would be expected when the total activation was similar when loading one muscle rather than two muscle groups, but this was not the case (Figure 3).

With the known force-velocity properties of muscle, some subtle differences in peak displacement would be expected if resistance to motion was purely impedance-based. This is due to the force-velocity curve being steeper when the muscle is stretched as compared to shortened.^32^ This was not observed, as peak displacement was greatest when co-contracting, with peak displacement being lowest when the shortened muscle was pre-loaded. Finally, kinematics from 0 to 50 ms post-perturbation were similar across all conditions (similar to our preliminary observations^11^) further arguing against impedance being the primary factor improving performance. While these observations argue against impedance being the only factor influencing performance, muscles do have passive properties that resist stretching,^32^^,^^45^^,^^46^^,^^47^ which would provide some contributions to limb control as previously speculated.^12^^,^^16^^,^^17^ The fact that 50 ms displacement was larger for our co-contraction as compared to the background conditions suggests individuals activated their muscles slightly less during co-contraction than for the “comparable” background loads. In spite of less initial muscle activity, performance was still generally better in the co-contraction condition.

Our study provides several observations that a key benefit of co-contraction on performance has a neural component. Even though maximal displacement was greater for even low levels of co-contraction, corrective responses to return and remain in the spatial goal were better than for comparable background load conditions. In contrast, there was inconsistency in corrective responses when resisting a background load. When the shortened muscle was pre-loaded there was target overshoot at all background load levels which compromised performance. When the stretched muscle resisted lower magnitudes of background loads there was similar poor performance with target overshoot. However, performance increased at the 5 Nm background load condition to be comparable to when co-contracting. These findings demonstrate that activating a single muscle group prior to perturbation can increase performance but is reliant on greater muscle activity levels. In contrast performance significantly improved from the relaxed to our 1.25 Nm co-contraction condition in 7/10 participants demonstrating that even low levels of co-contraction have significant improvements on motor performance. These neural-based benefits of co-contraction again supports our hypothesis that dual-control of agonist and antagonist muscle groups is likely an important benefit for control at least at low levels of muscle activity.

The associated patterns of EMG provide further insight on the potential neural benefits of co-contraction. Notably, increases in short- and long-latency muscle activity were observed when the stretched muscle was pre-loaded, which partially reflects gain scaling.^48^ Conversely, when the shortened muscle was pre-loaded prior to perturbation there was a reduction in R2 muscle activity. Across the protocol these general trends were observed in both stretched and shortened muscles, but only when that muscle was activated prior to the perturbation. These differences in spatiotemporal patterns are similar to previous observations when a single muscle was pre-loaded prior to perturbation.^49^^,^^50^^,^^51^

The influence of co-contraction on inhibitory control likely influenced performance. With simultaneous activation of opposing muscle groups prior to the perturbation, there are reductions in reciprocal inhibition^30^^,^^31^ allowing both muscles to contribute to control. Conversely, in the background load conditions, inhibitory signals would be sent to the unloaded muscles via Iα-interneurons,^52^ reducing their ability to contribute to the reactive response. Activity of both agonist and antagonist muscle groups prior to the perturbation would lessen this inhibition, allowing both muscles to contribute to a coordinated reactive response to improve performance.

The findings from experiment 3 reaffirm that reducing reciprocal inhibition by activating both muscle groups leads to performance increases. In this experiment the kinematics between the combination condition and reference background pre-load condition are consistent until approximately the time of maximum displacement. Following this time point even the non-equivalent activation of the opposing muscle groups contributes to increasing accuracy when returning to the target (i.e., comparing cyan and magenta and blue and green curves in Figures 6A and 6D).

Increased spinal feedback gains when pre-loading muscles have both potential benefits and detriments. The contributions of these spinal reflexes to limb control are apparent in the EMG data (Figures 5 and 7). Spinal reflexes are sensitive to load (i.e.,^21^^,^^22^^,^^23^^,^^24^^,^^25^^,^^26^^,^^27^) and are unable to deal with many contextual aspects necessary for goal-directed motor corrections.^53^^,^^54^ As revealed in the regression analyses, the influence of increased short-latency muscle activity generally had performance benefits (decreasing maximum displacement and reducing overshoot) across the range of muscle pre-activation conditions investigated. However, the exception to this trend was the positive association of stretched muscle activity to target overshoot when the shortened muscle was pre-loaded. With activity in the shortened muscle being suppressed there was little opposing muscle force to limit this overshoot, and in turn performance degraded.

An unexpected key finding in the present study is the substantial reduction in performance in the baseline (relaxed) condition when there was no co-contraction or background loads. Significant improvements in performance were seen with any level of muscle activity relative to when relaxed. With the robotic device providing weight support, very little (if any) muscle activity is necessary to maintain the index finger at the spatial target located in the middle of the workspace. Further, Figure 2 also highlights much greater trial-to-trial variability in maximal displacement in this baseline condition and may reflect small differences in pre-trial activity likely have large implications on resultant performance in these experimental paradigms. This interpretation is supported by greater short-latency activity in the stretched muscle being associated with reductions in maximum displacement (Tables S2 and S3) and the significant negative associations of pre-perturbation muscle activity to return times. Considering muscle activity, EMG responses were much slower in the baseline condition suggesting that motor corrective responses are greatly delayed when the motor system is not actively or minimally engaged. This could potentially explain performance differences beyond just being less motivated in the task.^55^ From a practical perspective this finding has important implications when considering results of previous studies in the motor control literature where pre-trial activity was not controlled (i.e.,^56^^,^^57^^,^^58^^,^^59^).

The present results highlight how levels of muscle activity in the limb can greatly impact motor performance. Co-contraction was demonstrated to help generate fast and accurate motor corrections, potentially explaining why we see its emergence in many difficult tasks. This ability to quickly and accurately correct errors or motion may also explain the paradoxical emergence of co-contraction in clinical populations such as patients with knee osteoarthritis^60^ or low back pain.^61^ While this co-contraction would increase joint loads, the benefits of rapidly and accurately responding to counter unexpected disturbances could be a beneficial tradeoff. Conversely, activating a muscle that is then shortened has performance detriments. This could have implications for injury risk in occupational settings, where pushing or pulling on an object that unexpectedly moves could lead to instability. However, this finding could be leveraged for practical benefits in athletic contexts where strategically forcing an opponent to shorten a pre-loaded muscle group in sports, such as wrestling or judo could have performance benefits.

### Limitations of the study

The current study was not without limitation. First, we draw conclusions about mechanisms of co-contraction influencing limb control, but it is challenging to investigate the contributions of impedance and neural mechanisms independently. The experimental conditions tested were done with the intention to make these inferences through systematically controlling muscle activity prior to the perturbation being applied.

A practical example showing the tradeoff of decisions made in choosing the analyses to conduct was to compare conditions when the net muscle activity was consistent but distributed differently across muscle groups opposed to comparing conditions with equivalent stretched muscle activity. This changes the impedance contributions to control because of known force-velocity relationships of muscle, as well as differences in stretched muscle impedance since short-range muscle stiffness is proportional to activation level. However, knowing that the shortened muscle would also contribute to the reactive response controlling the total amount of activation across muscle joints was what we believe to be the fairest comparison as practically this would pose the same metabolic demand. Finally, the experimental protocol in this study asks participants to knowingly generate consistent levels of muscle activation based on visual-feedback prior to applying the perturbations. This does not replicate the natural emergence of co-contraction observed when completing challenging tasks in our day-to-day lives. Future work should investigate whether the natural emergence of co-contraction offers similar performance benefits.

Finally, the study is limited by the sample size of 10 participants. However, a post-hoc power analysis confirmed the sample size was sufficient in size to achieve an observed power >90%. Even so, a greater sample could have been helpful in the experimental design considering some of the variability across participants in performance.

## Resource availability

### Lead contact

Requests for further information and resources should be directed to and will be fulfilled by the lead contact, Stephen H. Scott (steve.scott@queensu.ca).

### Materials availability

This study did not generate new unique reagents.

### Data and code availability

• De-identified human data have been deposited at OSF and are available at https://osf.io/ybcdx/?view_only=ab4d19416c7642f1924b889dfb7d8bc2. They are publicly available as of the date of publication.
• All original code has been deposited at OSF and are available at https://osf.io/ybcdx/?view_only=ab4d19416c7642f1924b889dfb7d8bc2 as of the date of publication.
• Any additional information required to reanalyze the data reported in this paper is available from the lead contact upon request.

## Acknowledgments

This work was supported by grants from the 10.13039/501100000038Natural Sciences and Engineering Research Council of Canada. D.P.A. was supported by a 10.13039/501100003321Queen's University postdoctoral award. S.H.S. was supported by a 10.13039/100004330GSK chair in Neuroscience. We thank Kim Moore and Helen Bretzke for their laboratory and technical assistance and the LIMB lab for helpful discussions.

## Author contributions

Conceptualization D.P.A., K.J.D., and S.H.S.; methodology D.P.A., K.J.D., and S.H.S.; software D.P.A.; validation D.P.A., K.J.D., and S.H.S.; formal analysis D.P.A.; investigation D.P.A.; resources S.H.S.; data curation D.P.A. and S.H.S.; writing – original draft D.P.A. and S.H.S.; writing – review and editing D.P.A., K.J.D., and S.H.S.; visualization D.P.A. and S.H.S; supervision S.H.S.; project administration D.P.A. and S.H.S.; funding acquisition S.H.S.

## Declaration of interests

S.H.S. is the co-founder and CSO of Kinarm, the company that commercializes the robotic device used in the present study.

## STAR★Methods

### Key resources table

**Table:** 

REAGENT or RESOURCE	SOURCE	IDENTIFIER
**Deposited data**		

Original Data	This paper	https://osf.io/ybcdx/?view_only=ab4d19416c7642f1924b889dfb7d8bc2

**Software and algorithms**		

MATLAB	Mathworks	https://www.mathworks.com/products/MATLAB.html
SPSS	IBM	https://www.ibm.com/products/spss-statistics
G∗Power	HHU	https://www.psychologie.hhu.de/arbeitsgruppen/allgemeine-psychologie-und-arbeitspsychologie/gpower

**Other**		

Kinarm Exoskeleton Lab	Kinarm	https://kinarm.com/kinarm-products/kinarm-exoskeleton-lab/
Delsys Trigno	Delsys	https://delsys.com/trigno/

### Experimental model and study participant details

#### Participants

Ten participants (sex = 4 ♀, 6 ♂; gender = 4 ♀, 6 ♂; stature = 1.74 ± 0.10 m; body mass = 76.4 ± 11.7 kg; age = 25.7 ± 3.2 yrs) completed the full study protocol. All subjects were neurologically healthy and gave informed consent prior to study participation. The experimental protocol was approved by the Queen’s University Research Ethics Board. No sex or gender effects on outcomes measures were observed. A within-subjects experimental design was used, so participants did not need to be allocated to experimental groups.

### Method details

#### Instrumentation

All experiments were conducted using a Kinarm Exoskeleton lab (Kinarm, Kingston, Ontario).^62^ The robotic device can apply joint-based loads to the elbow and/or shoulders. Participants were seated in an adjustable wheelchair while their arms were supported by plastic troughs attached to a mechanical linkage that permitted elbow and shoulder motion in the horizontal plane. The Kinarm lab has an integrated virtual reality system which can visually display objects aligned with the horizontal workspace and feedback of hand position. Vision of the upper limb was occluded with a physical barrier during the protocol. During all trials upper limb kinematics were sampled at 1000 Hz by the robotic system. Surface EMG was collected from the biceps brachii and lateral head of the triceps muscle groups at 1000 Hz (Delsys Inc., Boston, MA).

#### Experimental protocol

A target recapture task was performed in all experimental conditions where subjects began a trial by moving their right fingertip (position visualized with a white circle, 0.5 cm diameter) to a visual target (1 cm diameter) requiring 45° of shoulder flexion and 90° of elbow flexion (Figure 1). After a random wait time of 2–4 s, a mechanical perturbation was applied to either flex or extend the elbow joint (5 Nm magnitude, 10 ms ramp, 50 ms duration). The angle of the shoulder joint was controlled using the Kinarm motors to maintain the 45° angle for the duration of the trial. Once displaced from the start target visual feedback of the fingertip was removed, and participants were asked to reassume and hold the starting position as fast as possible. If the target position was reacquired within 500 ms and held for 1000 ms post-perturbation the trial was successful, and the target turned green to visualize success to the participant. Otherwise, the target color turned red to indicate an unsuccessful trial.

Pre-perturbation muscle activity was systematically varied prior to the mechanical perturbation. For both relaxed and co-contracting conditions, biofeedback of bicep brachii surface EMG was used to monitor pre-perturbation muscle activity, with perturbations only being applied once muscle activity was within a pre-defined range. EMG of the biceps brachii was monitored in real-time, with all data mean centred, rectified, and averaged in 100 ms sliding windows to provide real-time measures of muscle activity level. For co-contracting conditions the level of bicep brachii activity was specified to be within 20% of the level needed to resist either a 1.25, 2.5 or 5 Nm static elbow extensor load (these levels of muscle activity were identified prior to the main experiment in a postural load task when the participant maintained their finger at the spatial target). With no loads applied by the Kinarm, equivalent activity would need to be generated by the triceps brachii muscle group to maintain a static position, thus eliciting muscular co-contraction. While voluntary generation of co-contraction does not replicate its natural emergence in unstable environments (i.e.,^5^), these methods allow us to assess when different levels of co-contraction modify limb corrective responses. Similar methodology was employed for the relaxed condition, the perturbation was only applied if bicep brachii activity was below 20% of the activity needed to resist a 5 Nm static elbow extensor load.

In conditions where a background load was resisted prior to perturbation, loads were slowly applied (1000 ms ramp time) to either flex or extend the elbow to magnitudes of 1.25, 2.5 and 5 Nm. Perturbations were again applied after a random 2–4 s wait-time beginning once the full background load was applied and the fingertip location was in the specified target position. If the direction of the perturbation was the same as the applied background this is referred to as the stretched muscle pre-load conditions. If the directions were opposite, it was the shortened muscle pre-load condition. Finally, pre-perturbation activity was systematically modified to be a 2:1 ratio between elbow flexor and extensor muscle groups. This was done at two magnitudes, either having 5 and 2.5 Nm or 2.5 and 1.25 Nm of activation in the opposing muscle groups. This was achieved using a combination of background loads applied to the elbow by the Kinarm robot, and additional voluntary contraction monitored with biofeedback of bicep brachii muscle activity. In all pre-perturbation muscle activity conditions, the applied perturbation either flexed or extended the elbow joint. All trial types were randomized and interleaved. Ten trials were performed in each combination of pre-perturbation muscle activity and perturbation direction. Participants could self-select rest times throughout the protocol to minimize the likelihood of fatigue accumulation.

#### Data analysis

Trial success rate and completion time (time which target was recaptured and held for the following 1000 ms) was calculated as the primary dependent measure. Elbow angular displacement, with the absolute difference in elbow angle from the start position calculated for each trial, was retained as a kinematic dependent measure. Using the time-series elbow displacement data, we quantified maximum angular displacement, maximum target overshoot, and their corresponding times relative to perturbation onset. Finally, angular displacement 50 ms post-perturbation was calculated to quantify limb motion before neural feedback responses influenced limb motion.^63^

EMG data were mean centred and full wave rectified. EMG activity levels were normalized to the EMG levels needed to resist static 5 Nm elbow flexor or extensor loads for the triceps and biceps muscle groups respectively. Time-series normalized EMG are reported for all experimental conditions. Additionally, mean changes in EMG from pre-perturbation baseline activity were calculated for short- and long-latency reflex periods (R1 = 20–45 ms, R2 = 45–75 ms, and R3 = 75–100 ms) for both stretched and shortened muscle groups.^33^ Further, epochs were labelled as either ‘L’ or ‘S’ to refer to whether the data was specific to the lengthened or shortened muscle respectively. All EMG data were collapsed between the bicep and triceps muscle groups and labelled as stretched or shortened based on the direction of the applied perturbation (i.e., bicep activity in an extension perturbation and triceps activity in a flexion perturbation were collapsed as stretched muscle activity for a given pre-perturbation activity condition). All EMG and kinematic data analyses were completed using custom Matlab scripts (The Mathworks, Natick, USA).

### Quantification and statistical analysis

#### Sample size calculation

A post-hoc power analysis was conducted to compute the achieved power with the collected sample. With the effect size set to the lowest magnitude observed in a significant main effect (ꞃ^2^ = 0.421) and significance level of α = 0.05 the achieved power (1 – β) = 90.1.

#### Statistical analysis

Within each experiment, repeated one-way ANOVAs were used to test for differences in discrete kinematic and EMG outcome variables. A Holm-Bonferroni correction^64^ was applied to control for family-wise error across multiple ANOVAs with α set to 0.05. In practice all *p*-values <0.003 were deemed statistically significant. Post-hoc testing was conducted when significant main effects were observed. There were no significant differences in within-participant kinematic variability or performance outcomes across co-contraction and pre-load conditions supporting the appropriateness of considering participant means as dependent variables.

As a final comparison we correlated short-latency stretched muscle activity to both maximum displacement and target overshoot. Within each participant, trials within the same condition at different magnitudes (i.e., all co-contraction levels) were collapsed. For each of the co-contracting, muscle pre-load, and combination conditions the mean short-latency EMG activity in the stretched muscle was correlated to maximum displacement and target overshoot measured in the same respective trials. To determine trends across the sample the group means and standard deviations of r and unstandardized beta coefficients (β) are reported. All statistical testing was performed in SPSS (Version 29.0, IBM Corporations, Armonk, NY).

Throughout the manuscript asterisks (∗) are used to indicate significant differences in tables and figures reporting statistical outcomes.
