# Supplementary material for: Agonist-antagonist muscular co-contraction improves rapid corrective responses
Source: iScience. 2025 Jul 16;28(8):113122. doi: 10.1016/j.isci.2025.113122 (PMC12341523; doi:10.1016/j.isci.2025.113122)
Supplement: Document S1. Figures S1–S4 and Tables S1–S5 [file mmc1.pdf]

iScience, Volume *28*

Supplemental information

Agonist-antagonist muscular co-contraction improves rapid corrective responses

Daniel P. Armstrong, Kevin J. Deluzio, and Stephen H. Scott

Supplementary Materials

Supplementary Figures:

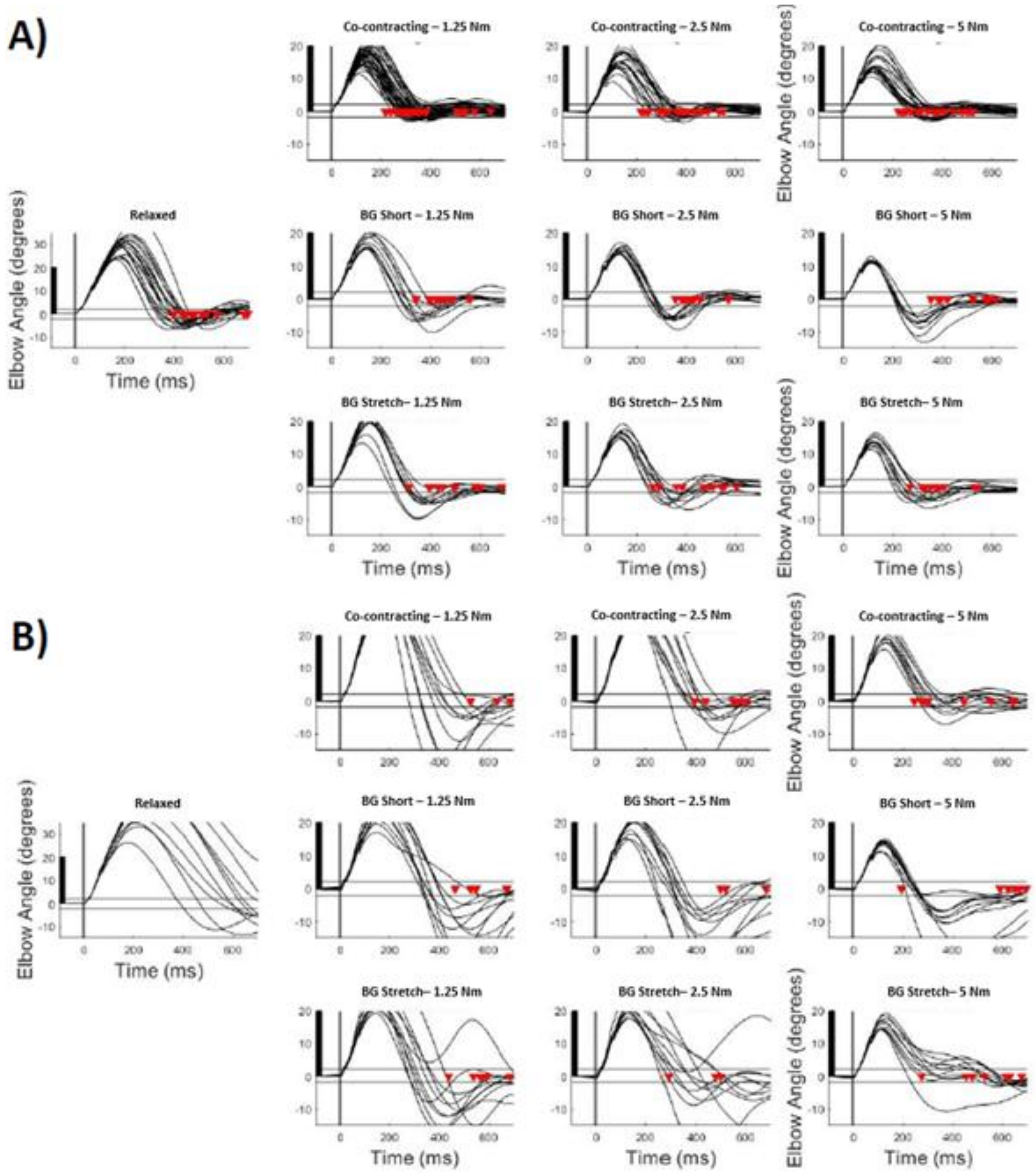

Figure S-1: Sample kinematics across all experimental conditions. Sample data are visualized for both a A) good and B) poor performer. Perturbation onset is denoted with the vertical grey line, and the successful target recapture range is visualized with horizontal black lines. Return times are shown with red arrows. Note: The y-axis scales differ between the Relaxed and remaining conditions. The black rectangles on each subplot are consistent in size to visualize differences in scale. Sub-plots are cut off at a y-axis value of 20 to maintain resolution across pre-loaded conditions.

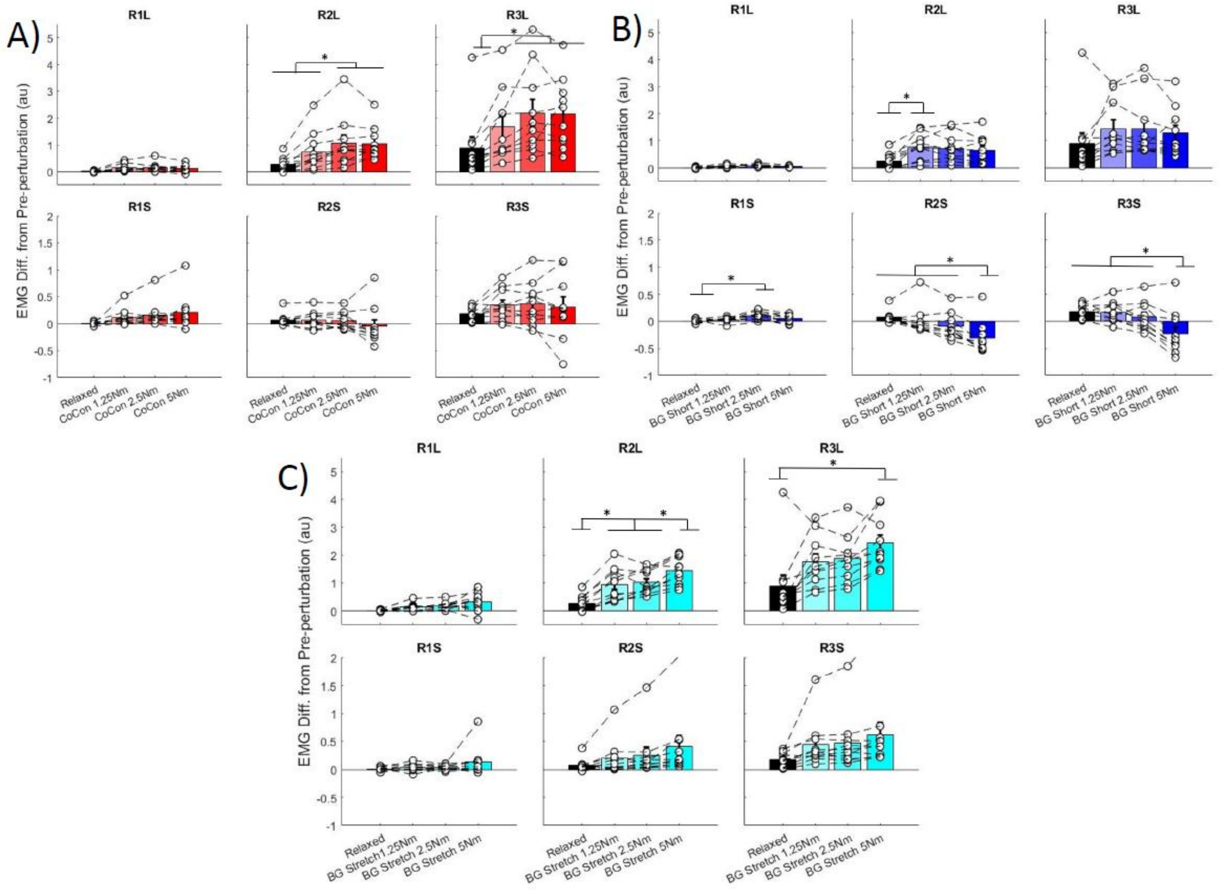

Figure S-2: Differences in EMG changes from baseline activity for the R1 (20-45 ms), R2 (45-75 ms) and R3 (75-105 ms) time epochs following perturbation for both lengthened (R#L) and shortened (R#S) muscle groups. Separate groups of subplots are shown for A) co-contracting, B) shortened muscle resisting background loads, and C) stretched muscle resisting background loads. All data are reported as mean +/- standard error.

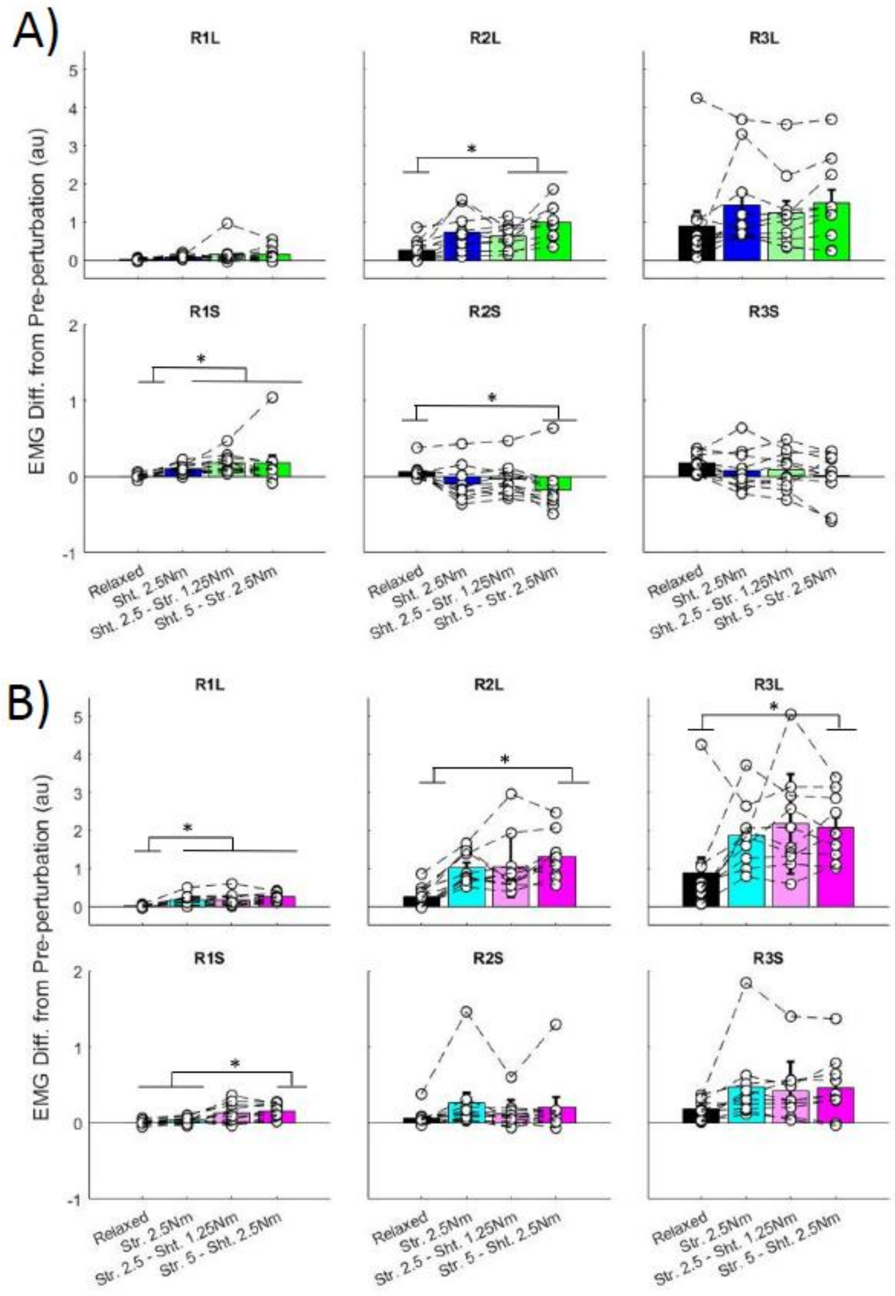

Figure S-3: Differences in EMG changes from baseline activity for the R1 (20-45 ms), R2 (45-75 ms) and R3 (75-105 ms) time epochs following perturbation for both lengthened (R#L) and shortened (R#S) muscle groups. Separate groups of subplots are shown for A) stretched muscle activity is greater than shortened muscle activity and B) shortened muscle activity is greater than stretched muscle activity conditions. All data are reported as mean +/- standard error.

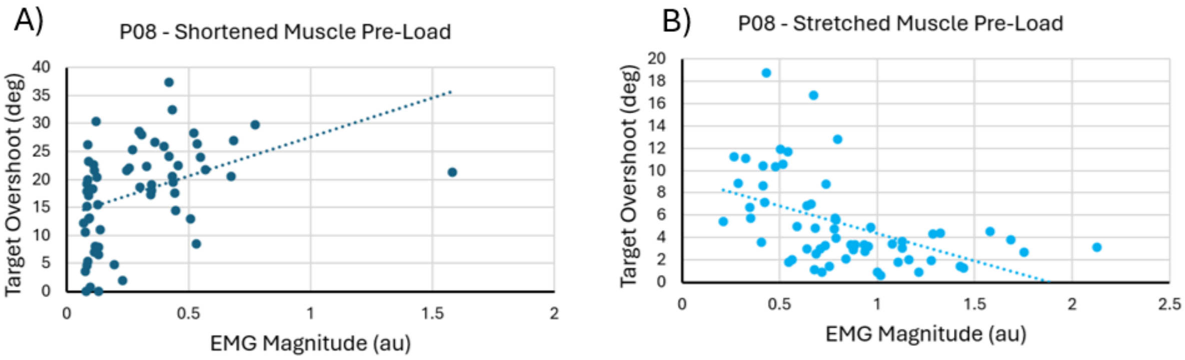

Figure S-4: Association of short-latency muscle activity in the stretched muscle to target overshoot. This is visualized both when A) shortened muscle is pre-loaded and, B) stretched muscle is pre-loaded for a single subject. Both plots show a significant association (shortened muscle pre-load $r = 0.404$; stretched muscle pre-load $r = 0.489$) but have opposite directionality.

Supplementary Tables:

Table S-1: Differences in return times across relaxed and co-contracting conditions within individual participants as measured by one-way ANOVAs.

	p value	Partial η^2	Post-hoc testing
P01	<0.001	0.437	Relaxed > All Co-con.
P02	<0.001	0.420	Relaxed > All Co-con.
P03	<0.001	0.213	Relaxed > All Co-con.
P04	<0.001	0.269	Relaxed > All Co-con.
P05	0.001	0.189	Relaxed > All Co-con.
P06	<0.001	0.409	Relaxed, Co-con.1.25 Nm > Co 2.5 & 5 Nm
P07	0.002	0.182	Relaxed, Co-con.1.25 > Co 2.5 & 5 Nm
P08	<0.001	0.313	Relaxed > All Co-con.
P09	<0.001	0.452	Relaxed, Co-con.1.25 > Co-con. 2.5 & 5 Nm
P10	<0.001	0.202	Relaxed > All Co-con.

Table S-2: Association (r) of Stretched muscle R1 response to maximum displacement across co-contracting, stretched pre-load, shortened pre-load and combination conditions. Asterisks (*) indicate significant correlations.

	Co-contracting	Stretched	Shortened	Stretch More	Short More
P1	0.612*	0.492*	0.237	0.448*	0.342
P2	0.218	0.636*	0.195	0.173	0.180
P3	0.379*	0.688*	0.450*	0.504*	0.319
P4	0.104	0.047	0.159	0.234	0.050
P5	0.414*	0.751*	0.083	0.708	0.455
P6	0.530*	0.343*	0.331*	0.352*	0.082
P7	0.520*	0.630*	0.100	0.503*	0.636*
P8	0.102	0.415*	0.302*	0.170	0.184
P9	0.668*	0.550*	0.254	0.533*	0.724*
P10	0.261	0.170	0.322	0.354*	0.189
**Average**	**0.380**	**0.472**	**0.243**	**0.397**	**0.316**

Table S-3: Unstandardized Beta Coefficient of Stretched muscle R1 response to maximum displacement across co-contracting, stretched pre-load, shortened pre-load and combination conditions.

	Co-contracting	Stretched	Shortened	Stretch More	Short More
P1	-5.13	-4.24	10.66	-1.97	-5.41
P2	-0.84	-3.55	-1.36	-0.53	-0.39
P3	-1.85	-3.14	-2.74	-2.70	-0.79
P4	-0.27	-0.46	1.21	-0.61	0.22
P5	-3.00	-2.83	1.06	-1.63	-2.36
P6	-10.00	-4.95	5.74	-8.14	0.94
P7	-19.69	-3.90	-3.48	-3.83	-15.06
P8	-1.01	-5.57	6.38	-2.21	-1.48
P9	-11.85	-9.81	-7.13	-6.04	-12.26
P10	2.50	4.15	-5.16	3.12	-0.86
**Average**	**-5.11**	**-3.43**	**0.51**	**-2.45**	**-3.74**

Table S-4: Association (r) of Stretched muscle R1 response to overshoot across co-contracting, stretched pre-load, shortened pre-load and combination conditions. Asterisks (*) indicate significant correlations.

	Co-contracting	Stretched	Shortened	Stretch More	Short More
P1	0.311*	0.257	0.367*	0.128	0.272
P2	0.223*	0.163	0.117	0.294	0.634*
P3	0.012	0.325*	0.345*	0.061	0.231
P4	0.021	0.165	0.002	0.084	0.164
P5	0.012	0.479*	0.313*	0.446*	0.205
P6	0.391*	0.472*	0.293*	0.219	0.068
P7	0.169	0.454*	0.135	0.158	0.245
P8	0.113	0.489*	0.404*	0.043	0.213
P9	0.409*	0.240	0.116	0.065	0.677*
P10	0.243	0.002	0.234	0.240	0.470
**Average**	**0.190**	**0.304**	**0.234**	**0.240**	**0.470**

Table S-5: Unstandardized Beta Coefficient of Stretched muscle R1 response to overshoot across co-contracting, stretched pre-load, shortened pre-load and combination conditions.

	Co-contracting	Stretched	Shortened	Stretch More	Short More
P1	-1.25	-2.80	21.60	-0.56	3.40
P2	-0.30	-0.70	0.74	-0.44	1.24
P3	0.23	-1.09	-1.97	0.193	-0.83
P4	-0.04	-2.39	-0.01	-0.59	-0.77
P5	0.03	-2.58	5.29	-0.78	-1.06
P6	-1.74	-3.72	4.63	-7.80	-0.54
P7	-6.00	-3.55	-6.87	-1.06	-4.46
P8	-0.43	-4.91	13.97	-0.85	-2.63
P9	-2.92	3.68	-3.83	-0.58	-11.80
P10	-1.26	0.01	-4.57	2.67	-2.91
**Average**	**-1.37**	**-1.80**	**2.89**	**-0.98**	**-2.03**
